# Serum erythropoietin concentration and its correlation with stage of diabetic retinopathy

**DOI:** 10.1186/s12886-019-1240-9

**Published:** 2019-11-14

**Authors:** Sofija Davidović, Nikola Babić, Sandra Jovanović, Sava Barišić, Desanka Grković, Aleksandar Miljković

**Affiliations:** 1 Faculty of Medicine, Department of Ophthalmology, University of Novi Sad, Novi Sad, 21000 Serbia; 2 Eye Clinic, Clinical Center of Vojvodina, Hajduk Veljkova 1-9, Novi Sad, 21000 Serbia

**Keywords:** Diabetic retinopathy, Growth factors, Erythropoietin

## Abstract

**Background:**

Erythropoietin (EPO) is one of the systemic angiogenic factors, and its role in ocular angiogenesis and in diabetic retinopathy (DR) is not yet fully understood. The latest research data reveal a possible correlation of higher erythropoietin concentrations in the blood and in the eye with the development of more advanced stages of DR.

The main aim of this work was to examine the possible influence of serum concentrations of erythropoietin on the development of diabetic retinopathy in patients with diabetes mellitus type 2.

**Methods:**

The research involved 90 patients examined at the University Eye Clinic of the Clinical Center of Vojvodina, Novi Sad, Serbia. The first group comprised 60 patients with diabetes mellitus lasting for 10 years or more, with diabetic retinopathy. The second, control group consisted of 30 healthy individuals. In the first group of 60 patients with diabetes, 30 of them had non-proliferative diabetic retinopathy (NPDR), and 30 had proliferative diabetic retinopathy (PDR). Laboratory EPO serum levels were determined, and they were correlated to the stage of DR. Concentration of EPO was assessed by ELISA method.

**Results:**

The highest average concentration of EPO in serum (9.95 mIU/ml) was determined in the group of people with diabetes with PDR. The lowest average concentration of EPO in the serum (6.90 mIU/ml) was found in the control group. The average concentration of EPO in serum in the group of patients with diabetes with NPDR was 7.00 mIU/ml. The EPO concentration in serum was elevated in the group of PDR, and it was directly proportional to the level of the clinical stadium of PDR, being significantly higher in the moderate and severe subgroup of PDR comparing to the control healthy subjects, NPDR and mild PDR (*p* = 0.007).

**Conclusions:**

Significantly elevated serum concentration of EPO in the advanced stages of DR, and positive correlation between EPO serum concentration and clinical stages of PDR, suggest that erythropoietin represents an important growth factor from blood, which plays a significant role in retinal ischemia and angiogenesis in diabetic retinopathy, especially in the proliferative stage of this disease.

## Background

Diabetes mellitus (DM) is a multi organic disease with a high incidence in the population, whose main cause is a carbohydrate metabolism disorder. One of the most common and at the same time, one of the most challenging chronic complications of DM is diabetic retinopathy (DR). Diabetic retinopathy leads to changes in the small blood vessels of the eye, and DR is nowadays considered as one of the leading causes of the impaired visual function and blindness, with significant socio-economic consequences on the working population which it affects [1].

Changed properties of blood vessels in DM and DR, lead to the increased liquid permeability which manifests as bleeding, oedema, and exudates in the eye. Another critical point in DM and DR pathogenesis is ischemia of the ocular tissues, which, through the production of vasoproliferative, angiogenic factors leads to neovascularisation - the growth of pathological blood vessels in the eye. These vessels have poor wall quality, and they will further lead to a vicious circle of new tissue ischemia and bleeding. The increased vascular permeability and pathologic neovascularisation are considered two major vascular pathogenic pathways for the development of diabetic retinopathy [2].

The development of DR has been studied for decades. It is well-known that local angiogenic factors in the eye play a dominant role, but recent studies, indicate the importance of systemic angiogenic growth factors, as well [3, 4]. One of angiogenic factors in our body is erythropoietin, and it is the body’s natural control mechanism for adjusting red blood cell count in response to the stressor. However, several important effects of EPO in the body are independent of its hematopoietic activity, and this is how EPO causes diabetic retinopathy changes [5, 6].

A growth factor is a substance that stimulates cell growth, proliferation, and differentiation. Most frequently these factors work as cytokines and hormones. The most important representatives of growth factors are epidermal growth factor (EGF), vascular endothelial growth factor (VEGF), granulocyte colony-stimulating factor (G-CSF), granulocyte-macrophage colony-stimulating factor (GM-CSF), insulin-like growth factor (IGF), erythropoietin (EPO), thrombopoietin (TPO), etc. [7]

Erythropoietin is a glycoprotein by its structure, which is composed of 166 amino acids. It is a pleiotropic cytokine, and a hormone with the function of circulatory growth factor [6, 8]. It is produced mainly in the kidneys, with only in a small part in the liver (10%); the primary stimulus for its releasing is tissue hypoxia. It acts by binding to transmembrane erythropoietin receptors (EPOR), which are primarily found on hematopoietic cells, but can also be found on the endothelial, myocardial, neural cells, and as well on the cells of liver, uterus, and retina [9, 10]. By binding to its receptor, erythropoietin activates physiological signalling cascade (including JAK 2 kinase signal-transducing, and STAT signalling cascade), as a response to hypoxia. There are recently discovered naturally occurring EPO mutations at specific activation sites of EPO, and this is how attenuation or modelling of EPO signalling in hematopoiesis leads to anaemia and other pathologic conditions [6]. Circulating erythropoietin primarily affects hematopoietic cells, and by stimulating angiogenesis, it is even considered as tumour-cells stimulator [11, 12].

There are theories about erythropoietic induction of neovascularisation caused by inflammation or ischemia. This induction occurs by mobilising the endothelial progenitor cells from the bone marrow, and thus increasing the number of cells in the circulation. From the existing capillaries and postcapillary venules, new blood vessels are being formed by degradation of the basal membrane of the blood vessel, migration of the endothelial cells and their mitosis, with the formation of bourgeons, lumens and vascular loops. All these steps represent angiogenesis that has to be distinguished from vasculogenesis [13]. Vasculogenesis occurs by the differentiation of endothelial cells from angioblasts. Angiogenesis can be physiological and pathological. Physiological angiogenesis represents a balance between proangiogenic and antiangiogenic factors in an organism, and occurs in the reproductive cycle, pregnancy and wound healing [14, 15]. Pathological angiogenesis occurs in neoplastic diseases leading to the acceleration of disease progression, and it is present in many other pathological conditions, like in diabetic retinopathy [16–18].

Numerous new studies indicate the main role of angiogenic growth factors on the development of proliferative diabetic retinopathy, and VEGF is considered to be the most important one [19], followed by erythropoietin, IGF-1, PDGF, etc. Intraocular synthesis of proangiogenic factors is in counterbalance with the production of antiangiogenic ones. It is considered that erythropoietin can have direct role in pathophysiology of diabetic retinopathy. However, there are still contradictory opinions about whether the role is aggravating or protective. It has been proved that erythropoietin significantly correlates with the origin of proliferative diabetic retinopathy [20]. In DM, and conditions of hypoglycemia and hypoxia, the increased number of erythropoietin receptors appear on retinal cells. Some authors consider this might be how retinal tissues survive in unfavourable conditions like in DM, inducing the increased binding of erythropoietin molecules [21]. In experimental rats with DM, intravitreal injection of EPO caused the increased number of erythropoietin receptors (EPOR) in neurosensory retina, with a protective effect against retinal neovascularisation and degenerative changes of photoreceptors [22, 23].

In certain researches it is found that the same EPO injection slows down retinal cells’ death and promotes the function of hematoretinal barrier. Therefore EPO is being considered as one of the new therapeutical options in the treatment of early DR and diabetic macular oedema [9, 24]. There is a recent discovery of naturally occurring nonhematopoietic variant of EPO hormone, which can activate only part of the signalling cascade, and therefore manifest only partial activation of EPO receptor, which could prove significant in the future regarding the application of new line drugs for new clinical conditions, apart from the already used ones.

However, there are different theories among the authors regarding the favourable influence of EPO on the progression of non-proliferative and proliferative DR. Some data show the improvement of DR by blocking of the production and effects of EPO in the eye [25].

Normal or low concentrations of erythropoietin are found in conditions of primary polycythemia, some erythropoietin-independent anaemias, but also in kidney-derived anaemia [26]. The importance of reduced concentration of erythropoietin is clinically confirmed in early diabetic nephropathy, as it resulted in anaemia which worsened diabetic retinopathy [27]. Also, in curing anaemia of renal origin, EPO given intravenously had positive effects on macular oedema, improved visual acuity in patients with DR and also led to the reduction of exudative maculopathy and proliferative changes of the disease [28, 29].

## Methods

This cross-sectional study included 90 examinees, over 50 years of age, 60 of them had verified diabetes mellitus (DM) and consequent ocular changes, and 30 of them served as control subjects. Patients were selected from the population of Vojvodina - Northern Serbia region, and all of the participants undertook detailed eye examination at the Eye Clinic of Clinical center of Vojvodina, and Medical faculty, Univerity of Novi Sad, in the period from 2009 to 2011. The study was conducted according to the principles of the Helsinki Declaration and approved by the written consent of the Ethics Committee of the Clinical Center of Vojvodina. Signed patient consent for participating in the study was obtained from all participants before the inclusion in the study, and it also included the section on their identification data publishing agreement.

The first group consisted of 60 consecutive patients suffering from DM type 2 who were treated for 10 years or more with oral, insulin or combined therapy, and who were referred to the University eye clinic for a complete eye examination, and evaluation of possible diabetic ocular fundus changes. The second group of 30 patients served as a control group of healthy subjects who were not diagnosed with any systemic disease or ophthalmic disease, except for the incipient senile cataract. The study did not include patients with an asymmetric finding on the eyes in terms of diabetic retinopathy, as well as patients with uncontrolled arterial hypertension, anaemia, diabetic patients with severe kidney damage, and patients on recombinant erythropoietin therapy.

The following data were recorded: name, surname, age, gender, and duration of diabetes mellitus. The height of the intraocular pressure was measured by the applanation tonometer, where the reference values ranged from 10 to 21 mmHg. Best-corrected visual acuity was determined by the Snellen chart and expressed in decimal values.

In the group of patients with diabetes, all patients had DM for more than 10 years. The presence of diabetic retinopathy and the progression of changes were determined. Patients with DR were further classified by ocular fundus changes, according to the existing international DR classification, into two main groups. First as a non-proliferative or proliferative DR group, and further into one of three subgroups within each main group, depending on the progression of changes - mild, moderate, and severe [30].

Laboratory analysis was performed at the Center for Laboratory Medicine of the Clinical Center of Vojvodina. Collected peripheral blood samples were labeled according to the standard protocols, transported and stored until the time of the laboratory analysis.

Individual blood samples (5 ml) were separated for the ELISA human erythropoietin immunoassay (Enzyme-Linked Immunosorbent Assay). They were frozen and stored at a temperature of − 20 C degrees within the hour of sampling and centrifugation until the analysis took place. EPO was measured by the commercially available human EPO ELISA commercial kit by the manufacturer R&D Systems Quantikine IVD for the quantitative determination of human serum or plasma erythropoietin. According to the manufacturer, medicines should not affect the accuracy of the results. The sensitivity of this test is very high, less than 0.6 mIU/ml. In addition, from blood samples of examinees, there were implemented laboratory measurements of the concentration of the glycated haemoglobin (HgA1c) in serum.

### Statistical analysis

All the data were statistically analysed using statistical software Statistical Package for Social Sciences - SPSS 17 and were presented graphically and in the tabular presentation. Descriptive statistics were used to describe the studied parameters. Differences in distributions of individual parameters between study groups were analysed using the parametric Student’s t-test, or the nonparametric Mann-Whitney test in case a distribution showed a significant deviation.

## Results

Table 1 shows the basic characteristics of the examinees, and ophthalmic clinical and laboratory data of the parameters that were analysed in the study. Out of the total number of examinees, there were 48 women and 41 men. The average age was 62.65 years (in the range from 50 to 81 years).

**Table 1:** Characteristics of patients in control group and in main groups with DM in relation to analyzed parameters

Patients		NPDR	PDR	Control group
Total number	90 (100%)	30 (33.3)	30 (33.3)	30 (33.3)
Male (%)	46.7	51.7	46.7	43.3
Female (%)	53.3	48.3	53.3	56.7
Age (mean ± SD, years)	62.0 ± 7.75	61.0 ± 7.13	64.67 ± 8.18	61.0 ± 7.72
Duration of DM (mean ± SD, years)	–	16.0 ± 7.36	16.0 ± 7.67	–
Mild	–	13.50 ± 7.93	20.5 ± 6.63	–
Moderate	–	16.0 ± 7.55	18.5 ± 6.29	–
Severe	–	18.0 ± 2.83	13.5 ± 9.57	–
HgA1c (%)		7.87 ± 1.15	8.03 ± 1.46	5.27 ± 0.64
BCVA (RE)		0.9 ± 0.26	0.45 ± 0.36	1.0 ± 0.05
BCVA (LE)		0.8 ± 0.26	0.35 ± 0.39	1.0 ± 0.18
BCVA (average for both eyes)		0.9 ± 0.23	0.45 ± 0.32	1.0 ± 0.1
IOP RE (mean ± SD mmHg)		16.0 ± 2.64	13.0 ± 3.27	14.0 ± 2.8
IOP LE (mean ± SD mmHg)		16.0 ± 2.37	14.0 ± 2.52	14.5 ± 2.95
IOP (mean ± SD mmhg) (average for both eyes)		16.0 ± 2.44	13.0 ± 2.73	14.25 ± 2.83

Legend:*DR* Diabetic retinopathy, *NPDR* Non-proliferative diabetic retinopathy, *PDR* Proliferative diabetic retinopathy, *DM* Diabetes mellitus, *HgA1c* Glycated hemoglobin, *BCVA* Best corrected visual acuity, *RE* Right eye, *LE* Left eye, *IOP* Intraocular pressure

All patients in the non-proliferative and proliferative DR group, were examined in detail with dilated pupils. They were further classified into one of three clinical stages (subgroups) of DR in each group- mild, moderate, and severe, according to the International Clinical Diabetic Retinopathy Disease Severity Scale – ICDRDSS (Table 2).

**Table 2:** International Clinical Diabetic Retinopathy Disease Severity Scale, American Academy of Ophtalmology

Proposed Disease Severity Level		Finding Observable upon Dilated Ophtalmoscopy
DM, without DR		No abnormalities
Non-proliferative DR	Mild	Microaneurysms only
	Moderate	Microaneurysms, exudate, venous beading, IRMA
	Severe	More than 20 intraretinal hemorrhages in each of 4 quadrants, definite venous beading in 2+ quadrants, prominent IRMA and 1+ quadrant; and no signs of proliferative retinopathy (4:2:1 rule)
Proliferative DR	Mild	Neovascularization Vitreous/preretinal hemorrhage
	Moderate	
	Severe - High-risk - Advanced	

The duration of diabetes mellitus in the NPDR group was 17.1 years (16 ± 7.36 years), and in the PDR group it was 18.13 (16 ± 7.76 years). There was no statistically significant difference in the duration of diabetes mellitus between the observed groups (*p* = 0.589).

The maximum duration of DM in the NPDR group was found in subgroups with an advanced form of illness18 ± 2.83 years, followed by subgroups of the moderate and mild form of NPDR. In the PDR group, the longest duration of DM was recorded in a subgroup of patients with a mild form of disease of 20.5 ± 6.63, followed by subgroups of moderate and severe PDR.

A statistically significant difference in the duration of DM between six analysed subgroups of patients was not determined (*p* = 0.707) in relation to the clinical stage of DR.

The value of glycated haemoglobin (HgA1c) was measured in all patients involved in the study. The average concentration of glycated haemoglobin in the blood of all patients is 7.15% ± 1.68%. A significant difference in the average level of glycated haemoglobin between the observed main patient groups was determined, *p* = 0.001. The highest average HgA1c value was observed in the group of patients with proliferative diabetic retinopathy (8.14%).

In the group of subjects with NPDR concentration, HgA1c was 7.96%, and in the control group 5.43%. A statistically significant difference was found between the average value of HgA1c in the control group and NPDR, *p* = 0.001, and between the control and PDR, *p* = 0.001 while a statistically significant difference was not found between the average values of HgA1c in the blood of patients with NPDR and PDR, *p* = 0.599.

The fundus of the eye was examined by direct or indirect ophthalmoscopy and biomicroscopy in artificial mydriasis. The control group comprised of subjects with no pathological changes in the retina, except vascular changes due to arterial hypertension, which were found in 67% of persons. The study did not include patients who were previously treated with laser photocoagulation, or who had any other eye treatment.

A statistically significant difference in mean visual acuity (BCVA) values were found between the observed groups for the right and left eye, *p* = 0.001, and for BCVA on average for both eyes together *p* = 0.001. The values of the best-corrected visual acuity BCVA were significantly lower in the PDR group (mean 0.44, median 0.45) compared to the values in patients with NPDR (mean 0.78, median 0.90) and the patients from the control group (mean 0.97, median 1.0) (Table 1).

The mean intraocular pressure (IOP) value, measured using applanation tonometrty in this study was 14.62 mmHg in the control group of the subjects, 14.84 mmHg in the group of NPDR, and 13.92 mmHg the PDR group.

A statistically significant difference was not found in the average IO*P* values between the observed groups for the right *p* = 0.347 and left eye *p* = 0.467, and for the IOP average for both eyes together *p* = 0.383.

**Fig. 1:**
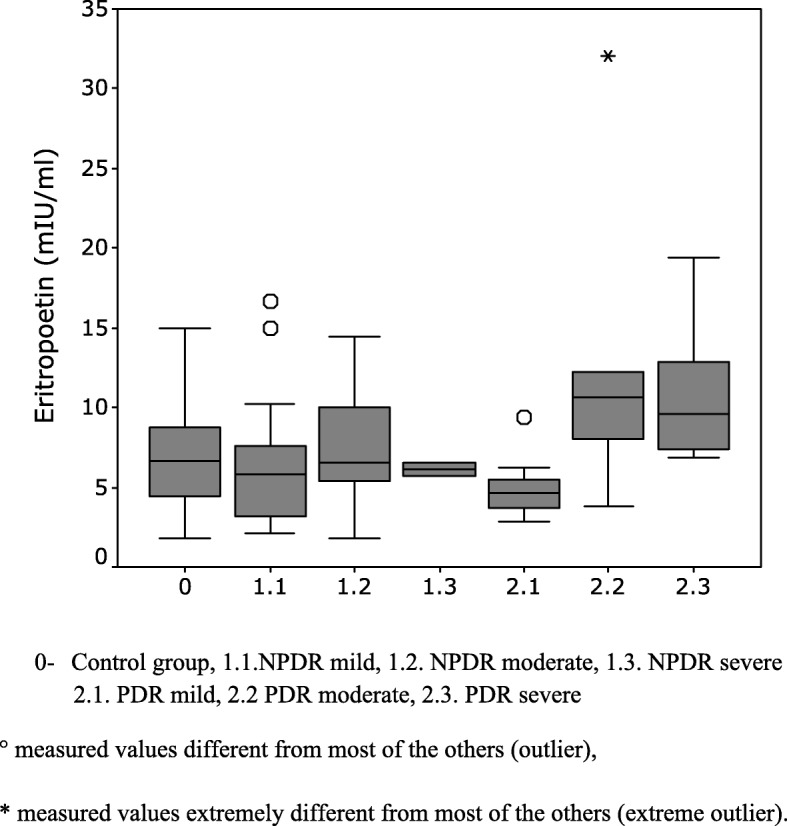
Erythropoietin concentration in blood (mIU/ml) in different clinical stages of nonprolipherative (NPDR) and prolipherative (PDR) diabetic retinopathy. 0.Control group, 1.1.NPDR mild, 1.2. NPDR moderate, 1.3. NPDR severe, 2.1. PDR mild, 2.2 PDR moderate, 2.3. PDR severe, ° measured values different from most of the others (outlier), * measured values extremely different from most of the others (extreme outlier)

Mean concentration of erythropoietin (EPO) in our analysed blood sample was 8.48 mIU/ml. The highest average EPO serum concentration was found in the PDR group of patients, 9.95mIU/ml. Serum concentration of EPO of 7.0 mIU/ml was noted in the NPDR group. The lowest EPO concentration was found in the control group of patients, 6.9mIU/ml (Fig. 1). The Mann-Whitney test did not show a statistically significant difference between the patients of the main observed groups - between the control group and the NPDR (*p* = 0.805), the control group and the PDR (*p* = 0.087), as well as among the subjects in NPDR and PDR (*p* = 0.071) (Table 3).

**Table 3:** Serum levels of erythropoietin (mIU/ml) in control group, in main groups and subgroups of analyzed patients with DM, with statistical difference

Epo concentration	NPDR	PDR	Control group
(mIU/ml)	7.00	9.95	6.90
Mild average (mean ± SD mmHg)	6.70 (5.8 ± 4.54)	4.97 (4.6 ± 2.05)	–
Moderate average (mean ± SD mmHg)	7.47 (6.5 ± 3.59)	13.55 (10.6 ± 10.88)	–
Severe average (mean ± SD mmHg)	6.10 (6.1 ± 0.57)	10.58 (9.6 ± 3.8)	–

Legend:*DR* Diabetic retinopathy, *NPDR* Non-proliferative diabetic retinopathy, *PDR* Proliferative diabetic retinopathy, *EPO* Erythropoietin

The mean EPO concentration in the NPDR group was 7.0mIU/ml. The highest concentration was recorded in the moderate NPDR subgroup (7.74mIU/ml), while the lowest was found in the subgroup of patients with the advanced NPDR (6.10mIU/ml). In the group of mild NPDR, erythropoietin level in serum was 6.70mIU/ml. With the Mann-Whitney test applied, a statistically significant difference in serum levels of erythropoietin between these three subgroups of NPDR was not found (*p* values of comparison of mild and moderate were *p* = 0.519, mild and advanced *p* = 0.933, and moderate and advanced *p* = 0.686.).

The average EPO concentration in the PDR group of patients was 9.95 mUI/l. By using the Mann-Whitney test, statistically significantly higher concentrations of EPO in the blood were confirmed in subgroups of patients with moderate (13.55mIU/ml) and advanced PDR (10.58mIU/ml), relative to the mild PDR (4.97mIU/ml) (*p* = 0.036 and *p* = 0.001, respectively) (Table 4). Whereas, there is no statistically significant difference in the serum EPO concentration between the two most severe forms of PDR, *p* = 1,000.

**Table 4:** Statistical difference of serum levels of erythropoietin (mIU/ml) in group of severe PDR compared to control group and subgroups of NPDR in patients with DM

*P* value	Mild NPDR	Moderate NPDR	Severe NPDR	Control group
Severe PDR	0.013^a^	0.035^a^	0.022^a^	0.004^a^

Legend: *DR* Diabetic retinopathy, *NPDR* Non-proliferative diabetic retinopathy, *PDR* Proliferative diabetic retinopathy, *EPO* Erythropoietin. ^a^Statistical significance

Statistically significantly higher concentrations of EPO in the blood were found in the subgroup of advanced PDR compared to control group (*p* = 0.004), but also each subgroup of NPDR, compared to mild NPDR (*p* = 0.035), moderate NPDR (*p* = 0.039), and advanced NPDR (*p* = 0.022) (Table 5).

**Table 5:** Statistical difference of serum levels of erythropoietin (mIU/ml) in moderate and severe PDR subgroup, compared to mild PDR subgroup in patients with DM

*P* value	Mild PDR
Moderate PDR	0.036^a^
Severe PDR	0.001^a^

Legend: *DR* Diabetic retinopathy, *PDR* Proliferative diabetic retinopathy, *EPO* Erythropoietin. ^a^Statistical significance

## Discussion

A common complication of diabetes mellitus is diabetic retinopathy which, in addition to cataract, glaucoma and senile macular degeneration, is one of the leading causes of visual impairment and blindness in the world [31, 32]. Although investigated in numerous studies for decades, the pathophysiological mechanism of the development of diabetic retinopathy has not been completely clarified. The eye, although a small organ, is by its metabolic properties most similar to the brain, and it is separated from the general circulation by a blood-ocular barrier. In the vitreous body, an increased concentration of vasoproliferative, angiogenic substances was found in patients with diabetes mellitus. However, it is not entirely clear whether this is a consequence of local production due to the presence of eye ischemia and/or the systemic impact of these factors on the eye and its metabolic processes. In explaining the onset of diabetic retinopathy nowadays, the increasing attention is also attributed to systemic angiogenic factors, and their possible ability to promote and increase diabetic vascular changes and occurring of neovascularization of the retina [33, 34].

Apart from vascular endothelial factor, as the leading factor, erythropoietin is one of the most prominent other representatives of systemic angiogenic factors, and its role in the occurrence of neovascularisation in the eye has not yet been entirely determined and understood. It plays a significant role as a growth factor in normal processes of erythropoiesis, by stimulating erythrocytes, their proliferation and differentiation, and the prevention of the apoptotic death of erythroid precursors with erythropoietin receptors [9, 13]. However, in numerous studies, the presence of erythropoietin receptors has been confirmed in various tissues such as kidney, liver, uterus, retina [10, 35]. In some studies, in diabetic patients, a significantly higher erythropoietin concentration in the vitreous body was found, which can be explained by the local production of erythropoietin in tissue hypoxia of the eye. This was true especially under the conditions of elevated blood sugar levels, in the presence of other cytokines in DR as well [19, 36].

Various studies have shown that erythropoietin affects endothelial cells the same manner as a vascular endothelial growth factor (VEGF), and for instance, in renal anaemia diabetic patients treated with human recombinant erythropoietin (rhEPO), rhEPO exhibits the same effect promoting increased intraocular angiogenesis and consequently impairment of DR [37, 38]. A statistically significant worsening of DR in the group of patients using rhEPO compared to the non-receiving group was also noted, and a direct proportionality of the serum erythropoietin concentration and the deterioration of diabetic eye disease was determined [39, 40]. Certain number of authors claim that the recombinant therapeutic EPO application allows better delivery of oxygen to ocular tissues, and the reduction of diabetic changes after its local or systemic administration [41, 42].

The role of EPO is still of interest for many researchers nowadays. It is still unclear whether erythropoietin in DR has a protective or aggravating role, comparing the results of various studies. Certainly, an increase in the number of erythropoietin receptors on retina cells in DR has been confirmed, which is considered to be a compensatory response to tissue hypoxia and hyperglycemia during DM. Under these conditions, the increased production and binding of EPO to erythropoietin receptors is the mechanism of survival of retinal nerve cells. It has been shown that erythropoietin inserted exogenously contributes to the preservation of the external blood-retinal barrier, acting on processes at the level of the retinal pigment epithelium. In addition, intravitreal injections of rhEPO have led to the inhibition of VEGF and stabilisation of the function of the blood-retinal barrier, and also to a short-term positive effect on the chronic macular oedema that has until then been practically refractory to any potential therapy [43].

During our study, we found that serum erythropoietin concentrations were increased in direct proportion with the severity of the clinical stage of proliferative diabetic retinopathy. The highest average value of erythropoietin in serum was found in the group of subjects with the most severe forms of proliferative diabetic retinopathy (9.95 mIU/ml). The lowest average concentration of EPO in the serum (6.90 mIU/ml) was found in the control group. The average concentration of EPO in serum in the group of people with diabetes with NPDR was 7.00 mIU/ml. There was no statistically significant difference in average EPO concentration within the main groups of healthy control subjects, and groups of patients with NPDR and PDR. EPO concentration in serum was markedly elevated in the group of PDR, and it was directly proportional to the level of clinical stadium of PDR, being significantly higher in moderate and severe subgroup of PDR comparing to controls, NPDR and mild PDR (h = 9.858, *p* = 0.007).

These data correspond to recent publications, where most elevated serum EPO concentration was determined along with the more advanced stages of diabetic retinopathy, like in the work of Semeraro, and Reida [44, 45].

The limiting factor of our study is the relatively small sample of 90 participants, and the lack of information on simultaneous intravitreal erythropoietin concentrations, correlated to the clinical stage of DR.

Furthermore, more comprehensive studies should also include simultaneous intravitreal and serum EPO concentration measurements, in order to obtain a more precise role of EPO in different stages of ischemic diseases, including diabetes mellitus, in order to find new possibilities for a better quality treatment and a better quality of life for patients with diabetic retinopathy.

## Conclusion

In our study a significantly higher concentration of erythropoietin in serum in severe forms of diabetic retinopathy was determined. We can conclude that erythropoietin represents one of the most important growth factors that, together with other angiogenic factors, like vascular endothelial factor, participates in ischemic and angiogenic processes in diabetic retinopathy, especially at its proliferative stage.

Further studies are needed, involving a larger number of subjects in a longer follow up period, to determine more precisely the effect of intravitreal and systemic erythropoietin and its concentration in diabetic retinopathy, in order to find new possibilities for better treatment and better quality of life of patients with diabetic retinopathy.

## Data Availability

The datasets used and analysed during the current study are available from the corresponding author, and available in supplement, as excel sheet file.

## Abbreviations

BCVA: Best-corrected visual acuity
DM: Diabetes mellitus
DR: Diabetic retinopathy
ELISA: Enzyme-Linked Immunosorbent Assay
EPO: Erythropoietin
HgA1c: Glycated hemoglobin
IOP: Intraocular pressure
LE: Left eye
NPDR: Nonproliferative diabetic retinopathy
PDR: Proliferative diabetic retinopathy
RE: Right eye
rhEPO: Human recombinant erythropoietin
VEGF: Vascular endothelial growth factor
